# Variation in type two taste receptor genes is associated with bitter tasting phenylthiocarbamide consumption in mature Targhee and Rambouillet rams

**DOI:** 10.1093/tas/txab142

**Published:** 2021-09-06

**Authors:** Kimberly M Davenport, J Bret Taylor, Dillan Henslee, Claire Southerland, Joel Yelich, Melinda J Ellison, Brenda M Murdoch

**Affiliations:** 1 University of Idaho, Department of Animal, Veterinary, and Food Sciences, Moscow, ID 83844, USA; 2 University of Idaho, Department of Animal, Veterinary, and Food Sciences, Idaho Agricultural Experiment Station, Nancy M. Cummings Research, Extension, and Education Center, Carmen, ID 83462, USA; 3 USDA, Agricultural Research Service, U.S. Sheep Experiment Station, Dubois, ID 83423, USA

**Keywords:** bitter taste, genetics, grazing, rangeland, sagebrush, sheep

## Abstract

Bitter taste perception in sheep can lead to avoidance of specific types of forage, such as sagebrush, which is present on many rangeland grazing systems in the Intermountain West. In humans, bitter taste perception is influenced by variation in several TAS2R genes, including more extensively studied *TAS2R38* and *TAS2R16*. We hypothesize that variation in taste receptor genes in sheep is associated with bitter taste. Therefore, the objective of this study was to examine variation in TAS2R genes in relation to consumption of a bitter tasting compound phenylthiocarbamide (PTC) which determines bitter “taster” and “non-taster” status in humans. Rambouillet and Targhee rams (*n* = 26) were offered various concentrations of PTC solution (0.2–12.29 mM) and water in a side-by-side presentation during two experiments. Blood was collected for DNA isolation and sequencing. Nineteen TAS2R genes were amplified and sequenced with long read Oxford Nanopore MinION technology. A total of 1,049 single nucleotide polymorphisms (SNPs) and 26 haplotypes were identified in these genes. Of these, 24 SNPs and 11 haplotypes were significantly (*P* < 0.05) associated with PTC consumption in *TAS2R3*, *TAS2R5*, *TAS2R8*, *TAS2R9*, *TAS2R16*, *TAS2R31-like*, *TAS2R38*, *TAS2R39*, and *TAS2R42-like*. Over 50% of the SNPs resulted in a change in amino acid sequence and several resided in potential regulatory regions, which could have downstream functional consequences and influence bitter taste perception in sheep. Further research is needed to validate these associations and elucidate the mechanisms that link variation in TAS2R genes to bitter taste perception in sheep. This may enable producers to select sheep more likely to consume bitter forage such as sagebrush as a flock and rangeland management strategy.

## INTRODUCTION

Dietary preference in humans is genetically associated with the 7-transmembrane G-protein coupled receptors known as type-two taste receptors (TAS2R) (Drewnowski and Rock, 1995). These are the only known taste receptors to perceive bitterness (Adler et al., 2000). Bitterness is the most sensitive of the five taste senses in sheep, often resulting in the avoidance of some forages (Goatcher and Church, 1970). Therefore, selection of sheep that could be genetically inclined to graze on less palatable and more bitter tasting vegetation could have economic and production applications in the areas of grazing management, although further research is needed for practical application.

*TAS2R38* and *TAS2R16* are among the variety of these genes responsible for bitter taste perception (Bufe et al., 2002; Kim et al., 2003). For instance, activation of *TAS2R16* receptors is initiated by bitter tasting β-glucopyranosides, such as salicylic acid (Bufe et al., 2002). The TAS2R38 receptors detect chemicals such as phenylthiocarbamide (PTC), which has been used extensively to test sensitivity to bitterness in humans and mice (Kim et al., 2003). While PTC is not a naturally occurring substance, the ability to perceive bitter tasting chemical compounds, such as PTC, has been correlated with the ability of animals to taste other bitter-tasting foods (Glanville and Kaplan 1965). In humans, PTC avoidance has specifically been linked to amino acid haplotype variations (PAV for “tasters” and AVI for “non-tasters”) on the *TAS2R38* gene (Kim et al., 2003).

Much like humans, rams have varying levels of sensitivity to PTC (Henslee et al., 2019). To date, there have been no studies conducted in sheep aimed at investigating the relationships between TAS2R genes and phenotypic traits, such as dietary preferences. Since *TAS2R38* and similar TAS2R genes are also found in sheep (Ferreira et al., 2013; Henslee et al., 2020), we hypothesized that there may exist similar genetic and phenotypic expression of bitterness avoidance. Thus, the purpose of this study was to evaluate the relationship between the consumption level of PTC and haplotype of various *TAS2R* genes within the ram.

## MATERIALS AND METHODS

### Animals and Phenotype Collection

All animal procedures were approved by an Institutional Animal Care and Use Committee (USDA, ARS, Dubois, ID) in accordance with the USDA, APHIS Animal Welfare Regulations (2013; 9 C.F.R. § 2.30−2.38 2013) and the Guide for the Care and Use of Agricultural Animals in Research and Teaching (FASS, 2010). Two trials were conducted at the USDA, ARS U.S. Sheep Experiment Station, Dubois, ID, in the spring of 2018. Phenylthiocarbamide (PTC) intake data was collected from yearling Rambouillet and Targhee rams (*n* = 26; *n* = 11 Rambouillet and *n* = 15 Targhee) where various concentrations of PTC solution (0.20–12.29 mM) and water were offered in a side-by-side presentation (Henslee et al., 2019). The experimental design, animal treatment, and animals were previously described by Henslee et al., 2019. Briefly, rams were housed in individual pens indoors and the environment was controlled at 10 °C with a 12 h light:dark cycle (Henslee et al., 2019). Feed, water and the treatment PTC solution were offered for ad libidum intake except between 1700 and 0700 h each day (Henslee et al., 2019). Rams were randomly assigned to pens within a breed and five treatments were assigned in a cross-over design (Henslee et al., 2019). The buckets with water and PTC solution were randomly assigned a location each time they were delivered (Henslee et al., 2019). Individual variation in the amount of PTC consumed was detected and rams clustered into low, medium, and high PTC consumption groups (Henslee et al., 2019). Blood was collected using EDTA tubes (BD Vacutainer EDTA tube, BD, USA) for DNA isolation post-treatment.

Classification of PTC consumption into high, medium, and low categories were calculated from total fluid intake (TFI) data (Henslee et al., 2019). In the absence of external influences, theoretically 50% of TFI should be consumed from each bucket (Goatcher and Church, 1970). Thus, classifications were formulated using a mean of 50% PTC consumption of TFI and divided into thirds, which resulted in three categories of equal range (low ≤ 16.6%; 16.6% < intermediate < 33.4%; high ≥ 33.4%). The number of rams that fell into the low, intermediate, and high categories was 8 (*n* = 5 Rambouillet and *n* = 3 Targhee), 10 (*n* = 4 Rambouillet and *n* = 6 Targhee), and 8 (*n* = 2 Rambouillet and *n* = 6 Targhee), respectively.

### DNA Extraction and Quantification

DNA was extracted from 500 µL of blood from each ram (*n* = 26) following the phenol chloroform method previously described and eluted in nuclease free water (Sambrook et al., 1989). Concentration and quality of DNA were determined with the Nanodrop Spectrophotometer (Thermo Fisher Scientific, Waltham, MA). All DNA extracted was above 20ng/ul in concentration and had a 260/280 ratio of >1.8 and 260/230 ratio of >1.9. Each sample was then diluted to 5 ng/µL with nuclease free water to perform PCR amplification.

### PCR Amplification of Target Genes

Amplification of target taste receptor genes in sheep was performed with FastStart High Fidelity PCR System, dNTPack (cat. 4738292001, Millipore Sigma, Burlington, MA) according to manufacturer’s instructions. Thermocycling conditions included an initial denature at 95 °C for 2 min followed by 35 cycles of denature at 95 °C for 30 s, annealing at 60 °C for 20 s, and extension at 72 °C for 40 s, followed by a final extension of 72 °C for 5 min. Amplicons were stored at 4 °C until sequencing. A list of genes, forward and reverse primers, and amplicon length which span the entire gene amplified are displayed in Table 1.

**Table 1.** Primers designed to amplify the entire family of known TAS2R genes in sheep

Gene	Gene Length (bp)	Forward primer*	Reverse primer*	Amplicon length (bp)**
*TAS2R3*	2841	CAGCTAACGGTCTGGAGGTC	CAGTAACAGCTTCACCGCCT	3111
*TAS2R4*	1710	CCCAGGTTCACTTTGGTGGT	CCACAGTCCTGCTGTTCCAA	2119
*TAS2R5*	2645	AGATTGCAGAAGGGTAAGACCA	TATCTCAAAACAGTCTCCTGACCAC	2807
*TAS2R7*	939	GGGACCGACAACTGCATTAC	TCCTCTGGCAGTTACTGTTAAGAT	1456
*TAS2R8*	929	GAGCTTGGAACTTTCGGAGGA	GTGCACTTTAGTAGGGGCCA	1399
*TAS2R9*	1164	TTTGAAGTCCCTGGCCAACA	TGGTGTGAAGTGTGAACGTGA	1500
*TAS2R10 (LOC101115110)*	930	AGGCATTCAGTCTGGGTGTG	GGGAGAAACCACTGGCAAGA	1398
*TAS2R10-like (LOC101122269)*	900	TGGAGGCATCTCTGTCAAGC	GGGAGAAACCACTCCAAGGG	1349
*TAS2R12 (LOC101114857)*	912	AGCAGTGGCGACACATACAT	TGAGAGGTCATCATCACTTCAGG	1180
*TAS2R16*	928	AGATGGCTGTGGGCAAAGAG	GGAACCTGGTCCCAAACTGG	1126
*TAS2R31-like (LOC101121003)*	914	TCCATCCCATAGTAGGGCAC	AGACACTTTTTGTTATTAGCTCAGG	1318
*TAS2R38*	1002	GTGGAAGGGCCCATTGATGTA	AGCTTCTGCATCACCCAAGG	1448
*TAS2R39*	1092	CACACCAGCGCATCCAAAAA	CAGCCCCGGAAATCTTGACT	1536
*TAS2R40*	1364	TAAACCGGGACTCTTGCCCT	TGACTCTGGGTTAGTGGGGT	1880
*TAS2R41*	936	GAGCTCAGTCACAGACACCC	TCCCAAAGGAGAAAGCCCAC	1391
*TAS2R42*	930	TGCCGATGATGAATGCACAC	GCCTCTTCTCCCAAATACGAGT	1519
*TAS2R42-like (LOC101120742)*	936	TGCCAGCACCAATGATGAGT	GGGCATGTCCAAATGATCGTG	1522
*TAS2R60*	954	AATTCATGGACAGGCAGCGA	TCTTTGGCCACATCAGGTCC	1438
*TAS2R67 (LOC101120486)*	939	AGTGGGCACATTCACTGCTT	TGATGCCAGTGATGCTTGCT	1416

* Gene specific primers do not include Oxford Nanopore forward and reverse sequence tags.

**Amplicon length was determined from sequence data.

### Library Preparation and Sequencing

Target gene amplicons were prepared for sequencing following the PCR Barcoding (96) Amplicons (SQK-LSK109) protocol available through the Oxford Nanopore community (www.nanoporetech.com). Barcoded amplicons were purified using MagBio HighPrep PCR magnetic beads (cat. AC-60005, MagBio Genomics, Gaithersburg, MD) according to manufacturer’s instructions. Amplicons were quantified with a NanoDrop Spectrophotometer, pooled in equimolar amounts, and subjected to end repair and prep and adapter ligation. Amplicons had a 260/230 ratio of >1.8 and 260/230 ratio of >2.0. A final cleanup was performed with AMPure XP magnetic beads (cat. A63880, Beckman Coulter, Indianapolis, IN) according to manufacturer’s instructions and quantified with a Qubit fluorometer (Thermo Fisher Scientific, Waltham, MA). The library was sequenced with a MinION device using one flow cell (R9.4.1; www.nanoporetech.com) for 48 hours.

### Bioinformatics and Data Processing

Base calling, demultiplexing by barcode, and trimming was performed with Guppy v3.2.2 (www.nanoporetech.com). The quality of FASTQ sequences were assessed with FastQC (https://www.bioinformatics.babraham.ac.uk/projects/fastqc/). Sequences were then mapped to *Oar_rambouillet_v1.0* with minimap2 v2.17 using default settings and indexed for visualization in the Integrative Genomics Viewer with SAMtools (Li et al, 2009; Li 2018; Salavati et al., 2020). Single nucleotide polymorphisms (SNPs) and haplotypes were identified in regions with greater than 100× coverage with marginPhase (Ebler et al., 2019). SNPs with a quality score <100 were discarded. Haplotypes were verified with visual inspection in the Integrative Genomics Viewer and manual examination of the sequence data (Robinson et al., 2011).

### Genetic Associations Analyses with PTC Consumption

Individual SNPs identified in the target genes for each animal were imported into the SNP & Variation Suite software v8 (Golden Helix, Bozeman, MT). Basic, genotypic, and additive genetic association tests were performed with the PTC consumption categories to capture the implied additive nature of this phenotype described previously and also investigate dominant and recessive associations with individual alleles and genotypes (Henslee et al., 2019). Haplotype associations with PTC categories were examined with a trend regression. Significance for SNPs and haplotypes was declared at *P* < 0.05. Variation in each gene was tested individually and independently.

## RESULTS

In total, 387,002 sequences were generated across all 19 TAS2R gene regions and 26 animals. A total of 1,049 SNPs were identified in these TAS2R gene target regions. A total of 24 SNPs were identified as significantly associated with PTC consumption category (*P* < 0.05) in seven TAS2R genes, specifically *TAS2R42-like, TAS2R31-like, TAS2R9, TAS2R8, TAS2R16, TAS2R3,* and *TAS2R5.* The precise genomic location of the SNP of each of the specific TAS2R genes are shown in Table 2. Significant SNPs were identified in the promoter 5’ and 3’ untranslated region of *TAS2R42-like, TAS2R9* and *TAS2R8*, respectively. All other associated SNPs were identified in the coding regions of the TAS2R genes. Of these, 9 SNPs were non-amino acid changing, 11 SNPs were amino acid changing and one induced a stop codon. The most significant (*P* = 0.002) SNPs that were identified were in *TAS2R16*.

**Table 2.** Significant SNPs in taste receptor genes associated with PTC intake in rams.

Position*	Gene**	SNP ID number (dbSNP)	SNP model	SNP P-value	Amino acid change
3: 218872939	upstream of *TAS2R42-like*	Novel	Genotypic	0.045	N/A
3: 218873800	*TAS2R42-like*	Novel	Genotypic	0.009	Ala/Ser
3: 218909947	*TAS2R31-like*	rs419195294	Basic	0.031	Asp/Gly
3: 219066474	*TAS2R9*	rs404321924	Basic	0.014	Gly/Asp
3: 219066528	*TAS2R9*	rs411528391	Basic	0.043	Asn/Ser
3: 219066529	*TAS2R9*	rs422591339	Basic	0.043	Asn/Asn
3: 219066621	*TAS2R9*	rs416718763	Basic	0.034	Pro/Pro
3: 219066682	*TAS2R9*	rs428638379	Basic	0.014	Ile/Val
3: 219066686	*TAS2R9*	rs403087052	Basic	0.007	Arg/Lys
3: 219068310	*TAS2R8*	rs412528176	Additive	0.034	Tyr/Tyr
3: 219068366	*TAS2R8*	rs423882545	Additive	0.034	Leu/STOP
3: 219068463	*TAS2R8*	rs404141749	Additive	0.034	Lys/Lys
3: 219068570	*TAS2R8*	rs426576409	Additive	0.010	Lys/Asn
3: 219068699	*TAS2R8*	rs416054395	Additive	0.010	Thr/Thr
3: 219068761	*TAS2R8*	rs410022040	Additive	0.010	Thr/Lys
3: 219069019	Downstream of *TAS2R8*	rs419056212	Additive	0.009	N/A
3: 219069021	Downstream of *TAS2R8*	rs401680429	Additive	0.009	N/A
4: 95494095	*TAS2R16*	rs420035590	Basic	0.002	Lys/Lys
4: 95494125	*TAS2R16*	rs400637145	Basic	0.002	His/His
4: 95494164	*TAS2R16*	rs412097853	Basic	0.015	Asp/Asp
4: 113926390	*TAS2R3*	rs425774520	Basic	0.023	Ser/Ser
4: 113927769	*TAS2R3*	rs413852493	Basic	0.013	Asp/Gly
4: 113928198	*TAS2R3*	rs415725026	Basic	0.004	Arg/Lys
4: 113956501	*TAS2R5*	rs422577624	Basic	0.048	Tyr/Cys

* Position is displayed as chromosome number: base pair location.

**The entire gene including upstream in the 5’ region and downstream in the 3’ region was amplified and sequenced to include coding and potential promoter and regulatory regions for each gene.

**Figure 1.**
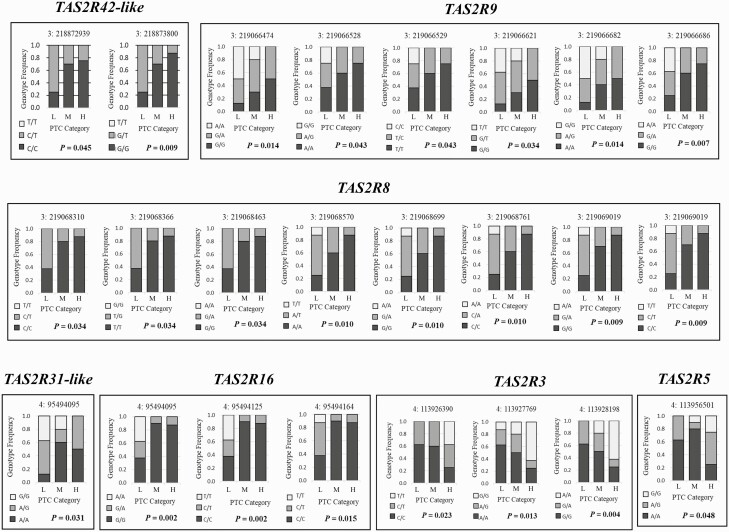
Genotype frequency in each PTC consumption category of low (represented by L), medium (represented by M), and high (represented by H) across SNPs significantly associated with PTC consumption category in *TAS2R42-like*, *TAS2R9*, *TAS2R8*, *TAS2R31-like*, *TAS2R16*, *TAS2R3*, and *TAS2R5*.

Next, we examined how each of the significant genotypes for each of the genes were associated with different amounts of PTC intake. Bar graphs for each significantly associated genotype were generated for low, medium and high PTC intake in order to gain a better understanding of the genetic relationship (Figure 1). In the *TAS2R42-like* gene, animals with C/C and G/G genotypes at 218,872,939 bp and 218,873,800 bp consumed more PTC. Whereas in the *TAS2R9* gene animals with A/A, G/G, C/C, T/T, G/G, and A/A genotypes consumed less PTC. The *TAS2R8* gene harbored the greatest number of significant SNPs, where animals with C/C, T/T, G/G, A/A, G/G, C/C, G/G, and C/C genotypes consumed more PTC. In contrast, *TAS2R31-like* only exhibited one significant SNP, where animals with a G/G consumed less PTC. Within the *TAS2R16* gene, which encompasses the two most significant SNPs, animals with an A/A, T/T, and T/T genotypes at these three locations consumed less PTC. Lastly, animals with a T/T, G/G, and A/A in *TAS2R3* significant SNP locations and a G/G in the single *TAS2R5* significant SNP consumed a greater amount of PTC.

Interestingly, animals with the alternative genotype at all of these loci exhibit increasing willingness to consume PTC in an additive manner. Although the significant SNPs in *TAS2R9*, *TAS2R31-like*, *TAS2R16*, *TAS2R3*, and *TAS2R5* had the most significant p-value in the basic genetic test, the additive genetic model was also significant (*P* < 0.05) for all of these SNP locations. In other words, the amount of PTC intake increased with heterozygosity and further increased in animals that exhibited homozygosity for the other SNP at all of the significant loci.

Several p-values of significant SNPs within the same gene were identical. This prompted us to examine if haplotypes in TAS2R genes harboring several SNPs were significantly associated with PTC consumption. A total of 26 haplotype blocks were identified across all genes, with 11 reaching significance and 9 containing independently significant SNPs (Table 3). The *TAS2R42-like* gene had the most significantly associated (*P* = 5.07E−05) haplotype with PTC consumption in a large block of 22 SNPs. The *TAS2R31-like* gene contained 12 SNPs in a haplotype block, with 1 SNP individually associated. Just over half of the SNPs in the significant *TAS2R9* haplotype block were independently associated with PTC consumption. Interestingly, the *TAS2R8* gene had two different significant haplotype blocks, with the most significant of the two (*P* = 0.007) the end and past the gene, with all four SNPs in the block also individually associated with PTC consumption. Significant haplotype blocks in *TAS2R16* and *TAS2R3* also contained SNPs individually associated with PTC consumption, whereas within in *TAS2R5*, only one SNP was individually significant. Three additional genes had significant haplotype blocks without independently significant SNPs: *TAS2R4*, *TAS2R38*, and *TAS2R39*.

**Table 3.** Significant haplotypes in taste receptor genes associated with PTC intake in rams.

Gene	Locations and haplotype*	P-value
*TAS2R42-like*	3:218872709, 3:218872798, 3:218872818, **3:218872939**, 3:218872942, 3:218872977, 3:218873004, 3:218873008, 3:218873059, 3:218873295, 3:218873381, 3:218873404, 3:218873413, 3:218873518, 3:218873552, 3:218873678, 3:218873703, 3:218873722, 3:218873771, **3:218873800**, 3:218873802, 3:218873869	5.067E−05
	G, T, A, **T**, C, G, A, G, G, C, T, T, T, T, G, C, C, G, G, **T**, G, A	
*TAS2R31-like*	3:218909211, 3:218909225, 3:218909242, 3:218909259, 3:218909471, 3:218909696, 3:218909813, 3:218909860, 3:218909873, 3:218909924, **3:218909947**, 3:218910086	0.004
	T, T, G, A, T, T, G, C, T, G, **A**, C	
*TAS2R9*	**3:219066474**, **3:219066528**, **3:219066529**, **3:219066621**, **3:219066682**, **3:219066686**, 3:219067174, 3:219067187, 3:219067247, 3:219067271, 3:219067323	0.002
	**G**, **A**, **T**, **G**, **A**, **G**, A, A, G, G, G	
*TAS2R8*	**3:219068310**, **3:219068366**, 3:219068393, **3:219068463**, **3:219068570**, 3:219068572, 3:219068634	0.030
	**T**, **G**, A, **A**, **T**, A, C	
*TAS2R8*	**3:219068699**, **3:219068761**, **3:219069019**, **3:219069021**	0.007
	**A**, **A**, **A**, **T**	
*TAS2R16*	**4:95494095**, **4:95494125**, **4:95494164**	0.009
	**G**, **C**, **C**	
*TAS2R3*	**4:113926390, 4:113927769**	0.028
	**T, G**	
*TAS2R4*	4:113940107, 4:113940522, 4:113941205, 4:113941293, 4:113941383, 4:113941740, 4:113941860, 4:113941862, 4:113941960	0.050
	C, G, G, C, C, T, C, G, T	
*TAS2R5*	4:113955710, 4:113956096, 4:113956118, 4:113956150, 4:113956190, 4:113956216, 4:113956230, **4:113956501**	0.043
	C, T, A, C, C, T, G, **A**	
*TAS2R38*	4:114099430, 4:114099567, 4:114099648, 4:114099679, 4:114099708, 4:114099738, 4:114099818, 4:114099916, 4:114100153, 4:114100217	0.027
	A, A, T, G, T, A, G, G, C, T	
*TAS2R39*	4:115407175, 4:115407221, 4:115407222, 4:115407601, 4:115407644, 4:115407656, 4:115407701, 4:115407842, 4:115407991, 4:115408072, 4:115408190, 4:115408410, 4:115408492	0.018
	T, T, G, A, C, G, G, A, T, T, T, C, T, C	

* Position is displayed as chromosome number: base pair location. Locations and nucleotides in bold are SNPs that are significant individually as well as within the haplotype.

## DISCUSSION

A total of 24 SNPs and 11 haplotype blocks in TAS2R genes were associated with PTC consumption in rams enrolled in this study. All SNPs significantly associated with PTC consumption except three were in coding regions of TAS2R genes, which is not surprising given that many of these genes are comprised of only a single exon. Of the significant SNPs, over half resulted in a change in an amino acid and one resulted in a stop codon, which may have downstream consequences related to the function of TAS2Rs and bitter taste perception. Upstream of *TAS2R42-like* also displayed a significant SNP, which is within the 5′ untranslated region (UTR) of the gene and likely contains a promoter. Two significant SNPs reside in the 3′ UTR region of *TAS2R8* which may also be a regulatory region. These changes in exon and regulatory regions may have significant influence on TAS2R function, however additional studies are needed to directly link these genetic changes to functional consequences.

The two most significant SNPs reside in *TAS2R16*, which has been found in humans to detect bitter tasting β-glucopyranosides, such as salicylic acid in willow bark, and may be important for sheep encountering similar substances in their grazing regime (Bufe et al., 2002). In addition, polymorphisms in *TAS2R3*, *TAS2R5, TAS2R9*, and *TAS2R31* have been linked to human intake of brassica vegetables, grapefruits and even the bitter undertones of artificial sweeteners (Mikołajczyk-Stecyna et al. 2020; Allen et al., 2013). This study suggests that more than one taste receptor gene potentially influences bitter taste perception as assessed by PTC but will need validation in a larger population of animals. Further, it is more difficult to characterize bitter taste perception in animals than it is in humans.

In addition to individual SNPs, haplotypes from 10 genes were associated with PTC consumption, including *TAS2R38* which also displays significant haplotypes (PAV and AVI) related to PTC taster status in humans (Kim et al., 2003). This gene exhibits 76.45% homology between sheep and humans (Henslee et al., 2020). Taken together with significant SNPs, these results suggest conservation of genetic influence on bitter taste perception across mammalian species. Interestingly, differences between the two breeds, Targhee and Rambouillet, were not detected however a larger dataset is needed to investigate any potential breed differences. Further research is needed to examine breed differences in the genetic relationship to bitter taste perceptions in sheep. In summary, this study identified several SNPs and haplotypes significantly associated with consumption of the bitter tasting PTC compound in sheep. This is important as genomic information could be informative in selecting sheep to graze specific forages available in the production environment. Further, sheep can serve as rangeland management tool by controlling overgrowth and weed-like behavior of shrubs which can lead to a reduction of rangeland plant diversity, carrying capacity, and wildlife abundance (Johnson et al., 1996; Launchbaugh, 2003). Sheep grazing can be utilized as a natural and practical alternative to other brush control strategies such as plowing, burning, and spraying when selected for specific management goals using genetic tools (Wambolt and Payne, 1986).

## IMPLICATIONS

This study identified variation in TAS2R genes significantly associated with consumption of the bitter tasting compound PTC. While PTC is not a naturally occurring substance, sensitivity to these compounds have been correlated with the perceived intensity of other bitter foods in mammals (Glanville and Kaplan, 1965). Understanding genetic relationships to bitter taste perception could have implications in grazing systems by enabling producers to select sheep that are more likely to consume bitter forages, such as sagebrush, and implement management strategies that compliment both sheep production and rangeland habitat.
